# The green roofs for reduction in the load on rainwater drainage in highly urbanised areas

**DOI:** 10.1007/s11356-021-12616-3

**Published:** 2021-02-01

**Authors:** Alicja Kolasa-Więcek, Dariusz Suszanowicz

**Affiliations:** Faculty of Natural Sciences and Technology, Institute of Environmental Engineering and Biotechnology, University of Opole, 6 Kominka Str, 45-032 Opole, Poland

**Keywords:** Storm water drainage, Urban areas, Forecasting, Green roof potential, Rainfall, Runoff

## Abstract

Rapid weather phenomena, particularly sudden and intense rainfall, have become a problem in urban areas in recent years. During heavy rainfall, urban rainwater drainage systems are unable to discharge huge amounts of runoff into collecting reservoirs, which usually results in local flooding. This paper presents attempts to forecast a reduction in the load on the rainwater drainage system through the implementation of green roofs in a case study covering two selected districts of Opole (Poland)—the Old Town and the City Centre. Model tests of extensive and intensive roofs were carried out, in order to determine the reduction of rainwater runoff from the roof surface for the site under study. The potential of the roofs of the buildings to make a green roof was also determined using geographical information systems (GIS), for a case study of two central districts of Opole. It proposed a methodology to determine the rainwater drainage system load reduction by making green roofs. The analyses carried out lead to the conclusion that, in the districts selected for the study, the execution of green roofs on 25% of the of buildings with the potential to implement this type of roof solution could reduce the load on the rain water system by a degree that protects the city area from local flooding.

## Introduction

Extreme weather events are undeniably occurring with greater frequency around the world, and Europe is no exception. In urban areas, heavy rainfall, sudden storms, and long periods of rainfall are causing more havoc, sometimes even to the point of posing a significant danger to the public. Most cities in Europe have separate sewerage systems designed specifically to handle heavy runoff, but most of these are underground and were built many years ago. Today, they do not have the capacity to efficiently handle the heavier runoff that so often obtains (Abd-Elhamid et al. 2020; Iglesias-Rey et al. 2017). In addition, in highly urbanised settlements, areas with very low rainwater permeability have an increasing share. This is usually due to high prices of building plots in cities and limited availability of land for investment. This limitation makes green areas very expensive and, sometimes, even impossible to create. In turn, this reduces the share of biologically active areas, which are permeable to rainwater, in city centres (Mentens et al. 2006; Palla et al. 2010) and is ultimately the cause of serious problems during periods of heavy rainfall, storms, or snowmelt.

The rainwater system is not able to drain large volumes of water in a short time. The excess water instead usually floods streets, other transportation routes, and basements of buildings (Hrudka et al. 2020; Voltarelli Franco da Silva et al. 2018). And that is the cause of serious problems during periods of heavy rainfall, storms, or melting of snow, because the rain water system is not able to drain large amounts of rainwater in a short time. The result is usually local flooding of street, transportation routes, and basements of buildings (Hrudka et al. 2020; Voltarelli Franco da Silva et al. 2018). In an even more difficult situation are urban areas that have only sewerage systems that collect surface runoff and wastewater for discharge into sewerage treatment plants. Any sudden increase in the surface runoff during torrential rain causes local flooding and paralyses the entire system. Such flooding is particularly dangerous because of the pollution in the wastewater. Alas, the situation cannot be corrected in the short term because it is at best very difficult and very expensive to increase the efficiency of the surface runoff system. This is mostly due to the high density of urban developments and the fact that the sewerage systems are routed deep underground (Abd-Elhamid et al. 2020).

As many authors have shown, green areas created on building rooftops offer a solution to this problem. They allow water to evaporate and be absorbed by soil and plants on the roof, thereby reducing the amount of water discharged into the rainwater sewers. This way only a part of rainwater body filters through the soil and drainage layers and is then discharged into the sewers (Baryła et al. 2018; Czemiel Berndtsson et al. 2009; Palla et al. 2010; Teemusk and Mander 2007; Van Seters et al. 2009). Of course, the effectiveness of this solution depends to a large extent on local climatic conditions (including, in particular, the volume and nature of rainfall, average temperatures, and humidity) and the vegetation that can be used on rooftops, as well as the density of buildings in urban areas. Various studies were carried out in Central Europe to determine the degree of retention of rainwater by the green roof or the impact of green roof layers on the quality of the runoff from the roof drainage system (Hubačíková et al. 2018; Pęczkowski et al. 2018; Pęczkowski et al. 2020).

Most of the research aimed only at establishing the rainwater retention rate for green roofs or at defining the percentage reduction of water runoff through roof layers (intensive or extensive) (Gettera et al. 2007; Harper et al. 2015; Stovina et al. 2012; Villarreal and Bengtsson 2005; Zhang et al. 2019). The most important issue for many authors was the possibility of storing water in layers of green roof (De-Villea et al. 2017; Poë et al. 2015; Zhang et al. 2019). Furthermore, most of the research was carried out in areas with conditions significantly different from those in Central Europe (Harper et al. 2015; Stovina et al. 2012; Zhang et al. 2019), and most often, the authors did not adequately consider the variability in daily rainfall and how it affects the efforts to reduce rainwater discharged from the green roof into the rainwater drainage system.

The city of Opole in Poland has been selected to conduct studies on how increasing the percentage of buildings with green roofs can reduce the outflow of runoff into rainwater sewerage systems in the conditions of Central and Eastern Europe. Opole is a medium-sized city with a population of 130,000 and is architecturally and climatically very representative of this part of Europe. The city has a separate rainwater sewerage system that empties directly into the Oder River, which flows through the city centre.

The case study of the city of Opole aimed to develop a methodology for determining the impact of green roofs on reducing the load on urban sewerage networks in areas with high variance in daily rainfall.

## Methods

**Fig. 1:**
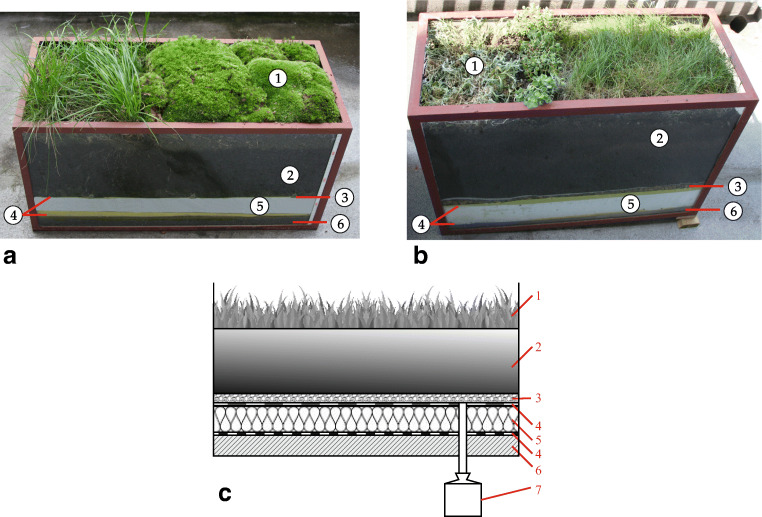
Model of extensive green roof used in research. **a** Extensive roof model, **b** intensive roof model, **c** diagram: 1—plants, 2—soil layer, 3—drainage layer, 4—vapour control layer, 5—insulation layer, 6—concrete slab, 7—measuring tank

In Opole, the first stage of research to realistically ascertain the reduction in the load on rainwater drainage in heavily urbanised areas by means of green roofs was to carry out studies with model roofs. Studies to determine the degree of reduction in the runoff of rainwater from the surfaces of green roofs were carried out on two model roofs that correspond respectively to the extensive (extensive roof model shown in Fig. 1a) and intensive roof structures (intensive roof model shown in Fig. 1b).

The roof models used in the study were glass tanks reinforced with steel frames, representing a 0.5-m^2^ green roof (as analyses indicate, this representative physical model is conducive to field study). For both models, the first layer was made of C16/20 class concrete. Then, the hydro-insulation layer was made in the form of PVC membrane. The next layer was thermal insulation made of polystyrene (in model 1—a layer of 8 cm thick extruded—XPS, and in model 2—a layer of 10 cm thick expanded—EPS). A drainage layer for each model was made: for model 1, in the form of drainage pipe, and for model 2, in the form of a 2-cm layer of gravel fraction of 1–1.5 cm (allowing excess rainwater to go to the vertical discharge pipe) (Suszanowicz 2018). For the extensive model, a layer of soil of 15 cm was used, and a layer of 35 cm was used for the intensive roof (Suszanowicz and Kolasa-Więcek 2019). In both cases, the soil mixtures were prepared in accordance with the recommendations of the German Landscape Research, Development, and Construction Society (Forschungsgesellschaft Landschaftsentwicklung Landschaftsbau—FLL) (Lösken et al. 2018; Kaiser et al.^2019^) and DAFA (Association of Flat Rooftops and Façade Contractors) (Lundholm 2015). Both models used the climate-specific vegetation of Central and Eastern Europe most common in the given type of roof: for the extensive roof model, grasses (*Poaceae*) and bryophyte (*Bryophyta*), and for the intensive roof model, grasses (*Poaceae*), perennials (*Herba perennis*), and sedum (*Sedum*). The rainwater from the drainage layer was discharged into the measuring tanks under each model (Fig. 1c). The roof models were set on the terrace on the top floor of a building located in the city centre.

An RG50 electronic rain gauge from SEBA Hydrometrie GmbH was set up by the models to record the daily amount of precipitation with an accuracy of 0.1 L/m^2^.

The months of May, June, September, and October were chosen for the experiment, which, as shown by the results of 10-year observations by the Opole weather station, have the highest daily rainfall (data available in the public domain www.imgw.pl—accessed April 2, 2019) (IMGW 2019). The readings of precipitation and volumes of water that accumulated in the measuring pools under the models were done once a day throughout all the months of the experiment.

The next stage of the research was analysis of roofs in urbanised areas. It was carried out for the case study of two selected districts of Opole—the Old Town and the City Centre. These districts have building density and geometric characteristics (building height, roof types, and slope) typical of medium and large cities in Central and Eastern Europe.

**Fig. 2:**
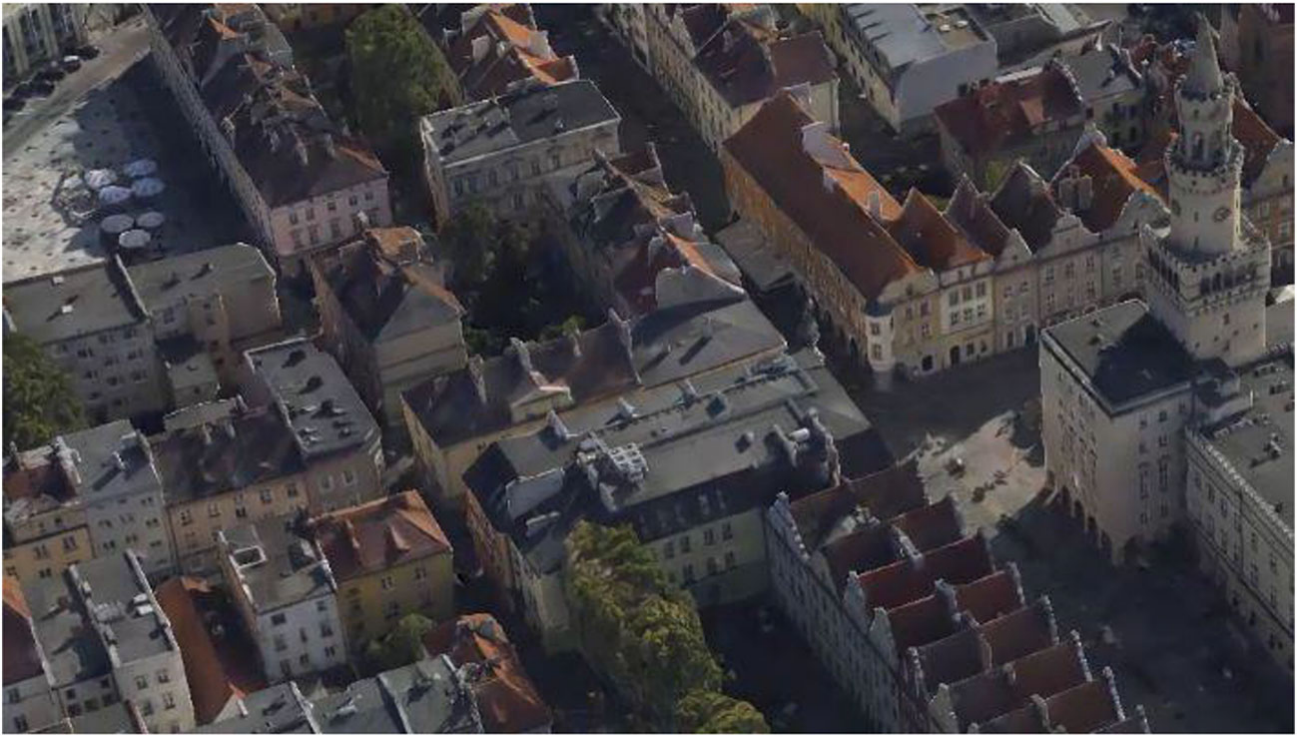
An example of an oblique aerial photograph of a section of the Old Town district (MGGP 2019)

The analysis of the potential of buildings for green roofing was carried out using geographical information systems (GIS), which are widely used for geographical area analysis. The arrangement of roofs in the area of the two administrative districts of the city of Opole was executed as follows:

1. A site survey was carried out for selected districts to identify the most common types of roofs. As a result of the site survey, 100 buildings architecturally representative of the area (50 in each district) were selected. Only single or double-pitch roofs were considered. They were flat or nearly flat roofs (with a slope of up to 5^o^) suitable for an intensive roof and roofs with a slope of up to 30^o^ suitable for an extensive roof.
2. The next part of the research work involved preliminary mapping of the field location of roofs on the working orthophotomap of the analysed districts of the city of Opole. ArcGIS then prepared an orthophotomap primer from ArcInfo v.10.6, setting the coordinate system as mapped, rectangular, PUWG 1992 (EPSG 2180). The orthophotomap was covered with a layer of buildings obtained from the basic map in order to precisely determine the locations of the buildings. On such a prepared base, screen vectorization of outlines of flat roofs or roofs with a pitch of up to 30^o^ was carried out, enabling their use in green roofs. The slope of the roof was analysed with the help of oblique aerial photographs of the Old Town and Downtown of Opole available at www.ukosne.pl (MGGP 2019). A representative photo of the Old Town is shown in Fig. 2.

3. A detailed field study was then carried out on the selected 100 buildings located in the test area to determine the average share of the active roof area, enabling biologically active material to be made (excluding from the roof area the space occupied by infrastructure elements such as chimneys, ventilation ducts, and roof hatches). In order to determine the suitability of a building for the construction of an extensive or intensive roof, the analysis took into account the slope of the roof, the load-bearing capacity of the building, and the infrastructure elements located on the roof surface. Also excluded were buildings designated as historical landmarks, of which the facades and roofing cannot by law be changed.

The final stage of the analysis was the development of a methodology to:

- determine the volume of the reduction of the outflow of rainwater from the surface of green roofs under the climatic conditions in the data,
- calculate the daily load on the rain water system in the analysed area, and
- forecast the degree of reduction of the rainwater load (in the case of rain with the highest observed intensity) by means of green roofs—extensive or intensive, with varying degrees of use of roofs of buildings that can accommodate such a solution.

## Results and discussion

**Fig. 3:**
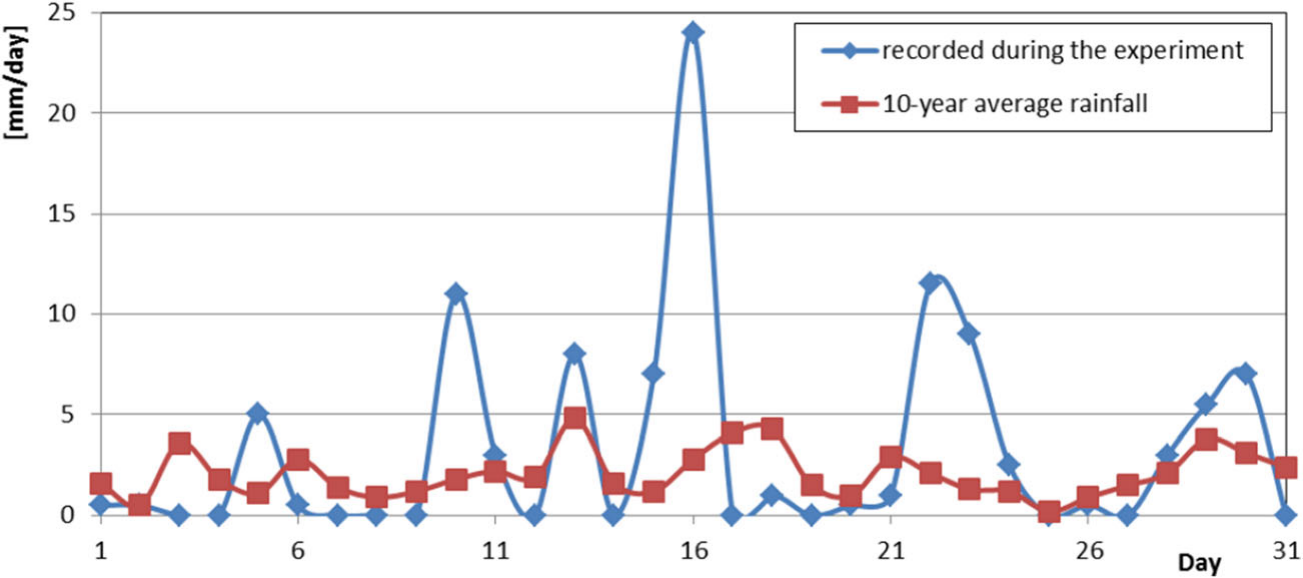
Recorded daily rainfall in May 2019 compared with the average daily rainfall determined through the 10-year observations

Observations made for the models of green roofs during the four selected months of 2019 (May, June, September, and October) showed that rainfall during the period of the experiment was highly variable. Sudden, very intense rainfall (most often observed in May and June) occurred during otherwise dry periods (especially long periods in September). The daily rainfall for most of the duration of the experiment differed significantly from the daily average rainfall values reported by the Opole weather station over a 10-year period. An example comparison of the daily rainfall in a selected month of the year (May 2019) with the average daily rainfall in May observed over the last 10 years is shown in Fig. 3.

As can be seen in Fig. 3, when designing a rainwater sewer network, the daily average rainfall values from multi-year observations cannot be the only premise. The current observations confirm that there is much greater variation in daily rainfall and, therefore, a need to reconfigure rainwater sewers in such a way that they can effectively discharge the large streams of wastewater which occur in the short time after torrential rain.

The studies also measured the relative humidity of the air directly above the vegetation covering the green roof models. On rainless days, it was found that the relative humidity of the air near the green roof is up to 5% higher than in the surroundings of the building where the models were located. This confirms the assumption that the rainwater absorbed by the layers of the green roof evaporates slowly over a long period time, which means more stable (more favourable to air quality) relative humidity of the air in the surroundings of the building.

**Fig. 4:**
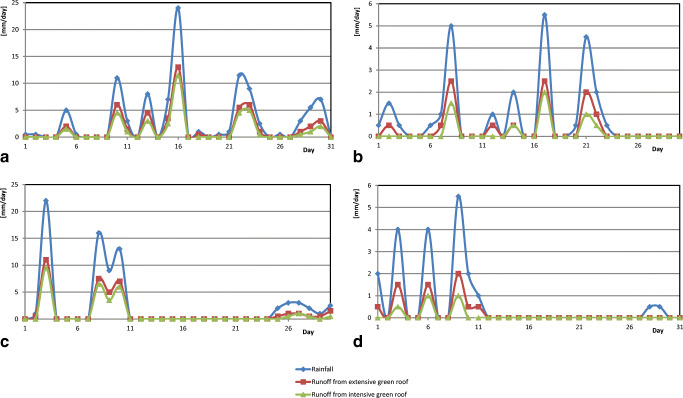
Recorded daily rainfall and outflows from green roof models for each month: **a** May, **b** June, **c** September, **d** October

The daily rainfall measured for specific months during the measuring period using a rain gauge, as well as the daily outflows from extensive and intensive roofs collected in the measuring tanks, are presented in graphs in Fig. 4.

As can be seen in the graphs shown in Fig. 4, the outflow of rainwater from green roof models is significantly lower than the recorded daily rainfall. Rainwater from rain not exceeding 5 mm/day on any given day is completely retained by both intensive and extensive roofs. Both during single-day rainfalls and rainfalls occurring over several consecutive days, intensive green roofs retain about 11% more rainwater than extensive ones. Observations have also found that, for rainfalls of several consecutive days, the degree of reduction in the outflow of rainwater from the green roof decreases by nearly 20%.

For the climatic conditions of Central Europe, the experiment allowed for determination of the extent that extensive roofs and intense roofs limits the runoff of rainwater into sewers. The results confirm the observations of other researchers that retention depends on the functionality of the growing media and the drainage layer (Kaiser et al. 2019). The results suggest that reduction coefficients for the outflow of rainwater from the green roof (RR) of 0.63 for the extensive roof and 0.74 for the intensive roof should be adopted. As many authors have shown in their research, intensive roofs reduce rainwater runoff into the sewerage system more than extensive roofs (Gettera et al. 2007; Harper et al. 2015; Stovina et al. 2012; Villarreal and Bengtsson 2005; Zhang et al. 2019). However, in contrast with the results from research in tropical climates and locations, rainwater retention by green roofs is significantly lower (Harper et al. 2015; Zhang et al. 2019). This is mainly due to the high variability of daily rainfall, as well as the typical vegetation used on green roofs in Central Europe. Following the observations of the variability in the value of rainwater runoff from green roof models, it was proposed to introduce a meteorological impact factor (CMC) that takes the values of:

- 0.7—for days with rain of more than 10 [mm/day],
- 0.8—for a minimum of 3 consecutive days of rainfall,
- 1—for precipitation after one rainless day, and
- 1.2—for precipitation after a minimum of 3 rainless days.

The proposed values of the CMC coefficient for correcting the predicted reduction in rainwater runoff from the green roof layers were determined after experimental research. In the case of heavy rain (more than 10 mm/day), the runoff reduction decreases by 30%. In the case of a minimum of 3 consecutive days of rainfall, the reduction in rainwater runoff decreases by 20%. For periods of at least 3 consecutive dry days, the rainwater runoff reduction increases by 20%.

In order to determine the reduction in the outflow of rainwater from the surface of green roofs (LDR) in the area under analysis, the following formula is proposed:

1 $$ \mathrm{LDR}={\sum}_i{A}_i\times {\mathrm{RR}}_i\times \mathrm{CMC}\times R\kern1.5em \left[L/\mathrm{day}\right] $$

where

- *i*—type of green roof: 1—extensive, 2—intensive;
- *A*—the surface of roofs of a given type in the analysed area;
- *R*—daily rainfall (mm/day).

A formula was also proposed to determine the daily load of rainwater drainage in the analysed area (LD), in the form of:

2 $$ \mathrm{LD}={A}_R\times R-\mathrm{LDR}\kern1.25em \left[L/\mathrm{day}\right] $$

where

- *A*_*R*_—the test area, equipped with a rain water sewer (m^2^).

**Fig. 5:**
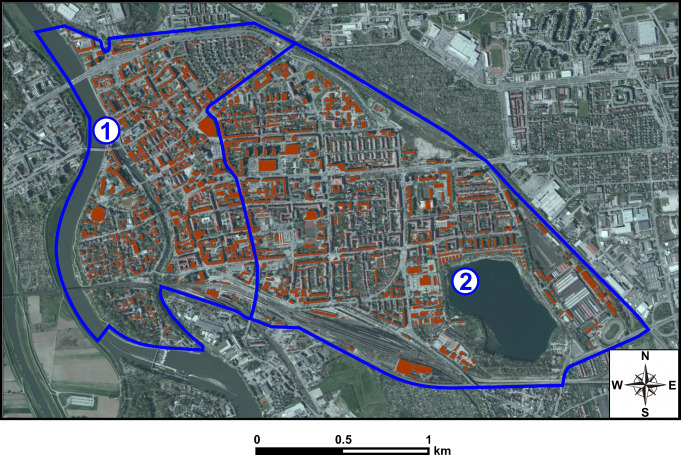
Distribution of buildings suggested for the green roofs in the area of districts: 1—the Old Town, and 2—the City Centre in Opole (Suszanowicz et al. 2019)

In order to determine precisely the area of the districts of the city of Opole (necessary for formula 2) and to determine the area of green roofs (necessary for formula 1), which can be performed in the analysed area, it is necessary to determine the number and area of buildings with the potential to accommodate a green roof. This requires the use of geographical information systems (GIS). The analyses indicate which buildings have such potential. For the purpose of the study, these buildings were marked in orange on the map shown in Fig. 5.

GIS studies allowed determination of the areas of both districts, excluding the area of the Oder River in the Old Town and the water reservoir “Piast” located in the City Centre. The designated areas are 1730.91 thousand m^2^ in the Old Town and 2901.73 thousand m^2^ in the City Centre. GIS analyses also identified the number of buildings with flat roofs or roofs with slopes of up to 30^o^ on which green roofs could be installed. Finally, after excluding designated landmark buildings, which cannot generally by law be altered, it was found that green roofs can be installed on 984 buildings in the Old Town and 1196 buildings in the City Centre.

Field studies of a selected representative group of 100 buildings located in the analysed districts of Opole show that 74% of the buildings indicated in Fig. 5 can accommodate extensive roofs and 26% intensive roofs. It was also found that, on average, 65% of the roof areas of these buildings could be biologically active. The percentage cannot be higher because of the need to leave technical infrastructures such as chimneys, ventilation duct outlets, and roof hatches on the roof surface.

The final result of this part of the study was the designation of roof surfaces of a given type in the area under analysis, substituted for the proposed correlation 1, which amounts to the *A*_1_ = 483.96 thousand m^2^ for extensive roofs and *A*_2_ = 170.04 thousand m^2^ for intensive roofs.

The daily rainwater load was calculated in the analysed area of the city of Opole using the proposed formula 2, with the highest recorded daily rainfall of 24 mm/day. In the subsequent calculations, the surface area of the green roofs was changed, starting from an area of 0 m^2^ and ending with areas of 483.96 thousand m^2^ for extensive roofs and 170.04 thousand m^2^ for intensive roofs. By referring the calculated values of the daily load of the rainwater system to the maximum load of the existing rainwater system in the city of Opole, it was found that the construction of green roofs on 25% of the available area of flat roofs or roofs with slopes of up to 30^o^ can reduce the load of the rainwater system enough to effectively prevent flooding in the area.

The impact of green roofs on reducing the load on rainwater drainage has already been analysed by various researchers (Hrudka et al. 2020; Mentens et al. 2006; Palla et al. 2010). However, the use of GIS systems proposed in this study to determine which buildings may be able to accommodate green roofs and to determine the impact of the variability of rainfall on the reduction of rainwater runoff from such roofs allowed for very precise forecasting of the reduction of load on urban rainwater drainage networks. This will be a significant factor in urban planning in that it will allow for reduction in the risk of local flooding, which occurs during heavy rainfall.

In the course of the field studies on the selected buildings, representative interviews were conducted with property managers regarding their interest in installing green roofs on their buildings. They generally expressed interest but also indicated that they lack adequate funds for such an investment. This points to the need to direct the attention of the national and local authorities to the introduction of support schemes for property owners who would like to install green roofs on their buildings. Such a solution would be beneficial for not only local sewerage networks but also for the urban environment in general, so they should be duly promoted and financially supported by the appropriate authorities.

A follow-up on the presented research has been planned on a reference green roof on one of the buildings of the University of Opole in the Old Town.

## Conclusions

Studies and analyses have shown that green spaces on the roofs of buildings in heavily urbanised areas can significantly reduce the load on the rainwater sewerage systems of cities in the climatic conditions of Central Europe. About 44% of the rainwater from torrential rains is retained in the layers of the green roof, and in the case of rains not exceeding 5 mm/day, rainwater is completely retained by both intensive and extensive roofs. The results confirm the observations of other researchers that retention depends on the functionality of the growing media and the drainage layer (Kaiser et al. 2019). Observations by researchers in other Central European cities have also been corroborated, namely, that the optimal selection of vegetation used on a green roof strongly depends on local climatic conditions (Hrudka et al. 2020; Pęczkowski et al. 2018; Pęczkowski et al. 2020).

The model studies carried out on green roofs, extensive and intensive, allowed for determination of the reduction of the outflow of rainwater from the roof surface located in an area representative of Central Europe. The designated values for the coefficient of reduction of the outflow of rainwater from the green roof (RR) are 0.63 for the extensive roof and 0.74 for the intensive roof.

It is proposed to include a meteorological impact factor (CMC) taking into account the volume of daily rainfall R and the occurrence of consecutive rainfall on consecutive days. The proposed values of the CMC coefficient for correcting the predicted reduction in rainwater runoff from the green roof layers were determined after experimental research. In the case of heavy rain (more than 10 mm/day), the runoff reduction decreases by 30%. In the case of a minimum of 3 consecutive days of rainfall, the reduction in rainwater runoff decreases by 20%. For periods of at least 3 consecutive dry days, the rainwater runoff reduction increases by 20%.

The proposed methodology for determining (using GIS systems) the potential of buildings to accommodate green roofs can be applied in heavily urbanised areas, not only in Central Europe but also in other temperate climate locations. This means quick forecasting of the reduction of the load on the rainwater drainage systems when installing a different number of green roofs on buildings located in the area under analysis.

The study concluded that, for the area under analysis, the construction of green roofs on 25% of buildings that could accommodate this type of roof solution could reduce the load on the rain water system sufficiently to prevent local flooding.

Further studies should be carried out on the impact of the green roof layers on the reduction of rainwater runoff in heavily urbanised areas. A reference green roof is planned on one of the buildings of the University of Opole, in the Old Town. The roof will be made with a wider variety of vegetation, which will increase the evaporation of water stored in the soil layer (water-storing admixture will be used in the soil) and boost the city’s biodiversity.

## Data Availability

All data generated or analysed during this study are included in this published article. This manuscript has not been previously published, and the paper is currently not under consideration by another journal. All authors have approved of and have agreed to submit the manuscript to this journal.
